# Zic family member 5 promotes RIO kinase 3 expression to enhance pancreatic cancer survival

**DOI:** 10.1111/febs.70125

**Published:** 2025-05-03

**Authors:** Reiko Satow, Yuki Kashiwaba, Misaki Okao, Shin Takano, Yuna Aiga, Atsuko Yoneda, Kazuyoshi Hosomichi, Kiyoko Fukami

**Affiliations:** ^1^ Laboratory of Computational Genomics Tokyo University of Pharmacy and Life Sciences Hachioji‐shi Japan; ^2^ Laboratory of Genome and Biosignals Tokyo University of Pharmacy and Life Sciences Hachioji‐shi Japan

**Keywords:** cancer survival, drug resistance, gemcitabine sensitivity, pancreatic ductal adenocarcinoma

## Abstract

Pancreatic ductal adenocarcinoma (PDAC) is one of the most lethal malignancies with few effective therapies available. We previously determined the essential role of Zic family member 5 (*ZIC5*) in the survival of PDAC cells. In this study, we showed that targeting *ZIC5* can effectively shrink PDAC tumors treated with gemcitabine *in vivo* and investigated the molecular mechanisms involved. When tumor‐bearing mice were injected intravenously with *ZIC5*‐targeting small interfering RNA, tumor volume was significantly reduced by gemcitabine treatment. RNA‐sequencing analysis was used to identify the genes affected by *ZIC5* knockdown. Among these, we selected the genes whose mRNA expression levels correlated with that of *ZIC5* in pancreatic cancer and those associated with poor prognosis in patients with pancreatic cancer. Further analysis revealed that RIO kinase 3 (*RIOK3*) promotes PDAC cell survival, whereas *ALDH3B1*, *PTGES*, and *TUFT1* contribute to gemcitabine resistance in MiaPaca‐2 cells. We identified *RIOK3* as a direct target gene of ZIC5 using ChIP and luciferase assays. Furthermore, stable expression of RIOK3 in PANC‐1 cells reversed the reduction in cell number following *ZIC5* knockdown. These findings highlight RIOK3 as a critical target of ZIC5, which is involved in survival signaling in PDAC cells.

## Graphical abstract

This study demonstrates that targeting ZIC5 increases gemcitabine sensitivity and induces pancreatic cancer shrinkage *in vivo*. The findings also show that ALDH3B1, PTGES, and TUFT1, the downstream genes of ZIC5, contribute to gemcitabine resistance in pancreatic cancer cells. Furthermore, RIOK3 is predicted to be an important direct target of ZIC5, which promotes survival signaling in these cells.

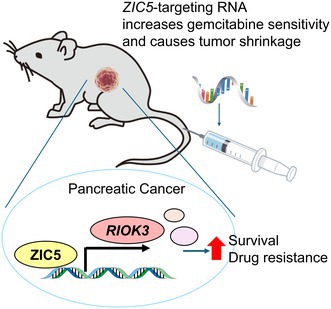

**Abbreviations**
MDSCs: myeloid‐derived suppressor cells
PDAC: pancreatic ductal adenocarcinoma
RIOK3: RIO kinase 3
STAT3: signal transducer and activator of transcription 3
STR: short tandem repeat
TAMs: tumor‐associated macrophages
TCGA: The Cancer Genome Atlas
TPM: transcripts per kilobase million
Tregs: regulatory T cells
ZIC5: Zic family member 5

## Introduction

Despite advances in the understanding of the biology of pancreatic ductal adenocarcinoma (PDAC), its clinical outcomes remain poor [1]. Therefore, there is an urgent need for the development of effective molecular therapeutic agents against PDAC.

Previously, we had identified Zic family member 5 (ZIC5) as a critical survival transcription factor in melanoma, colorectal cancer, prostate cancer, cholangiocarcinoma, and PDAC cells [2, 3]. *ZIC5* expression is enhanced in patients with various types of cancer and is related to poor prognosis, whereas *ZIC5* expression is barely observed in most normal human adult tissues [3]. ZIC5 contributes to the activation of the signal transducer and activator of transcription 3 (STAT3), which promotes the expression of anti‐apoptotic factors. Thus, ZIC5 enhances survival signaling and drug resistance in various types of cancer [3, 4, 5].

ZIC5 promotes the malignant phenotype of hepatocellular carcinoma (HCC) by promoting β‐catenin signaling and COL1A1 expression [6, 7]. In nonsmall‐cell lung cancer, *ZIC5* knockdown causes cell cycle arrest and the downregulation of both CCNB1 and CDC25C [8]. However, the direct molecular targets of ZIC5 in these tumors are unknown. Further, in colorectal cancer cells, it is shown that ZIC5 forms a complex with TCF7L2 and β‐catenin to act as a transcriptional repressor of *SLC2A1* [9]. ZIC5 also directly regulates *CDH1* expression in melanoma [4]. However, as mentioned in the case of HCC, the direct target molecules of ZIC5 in PDAC have not yet been identified.

In this study, we investigated genes directly targeted by ZIC5 in PDAC cells and identified one of the direct target genes as important for PDAC survival. Furthermore, we showed that targeting *ZIC5* effectively reduced the size of PDAC tumors treated with gemcitabine *in vivo*.

## Results

### Targeting *ZIC5* increases gemcitabine sensitivity and causes PDAC shrinkage *in vivo*

**Fig. 1:**
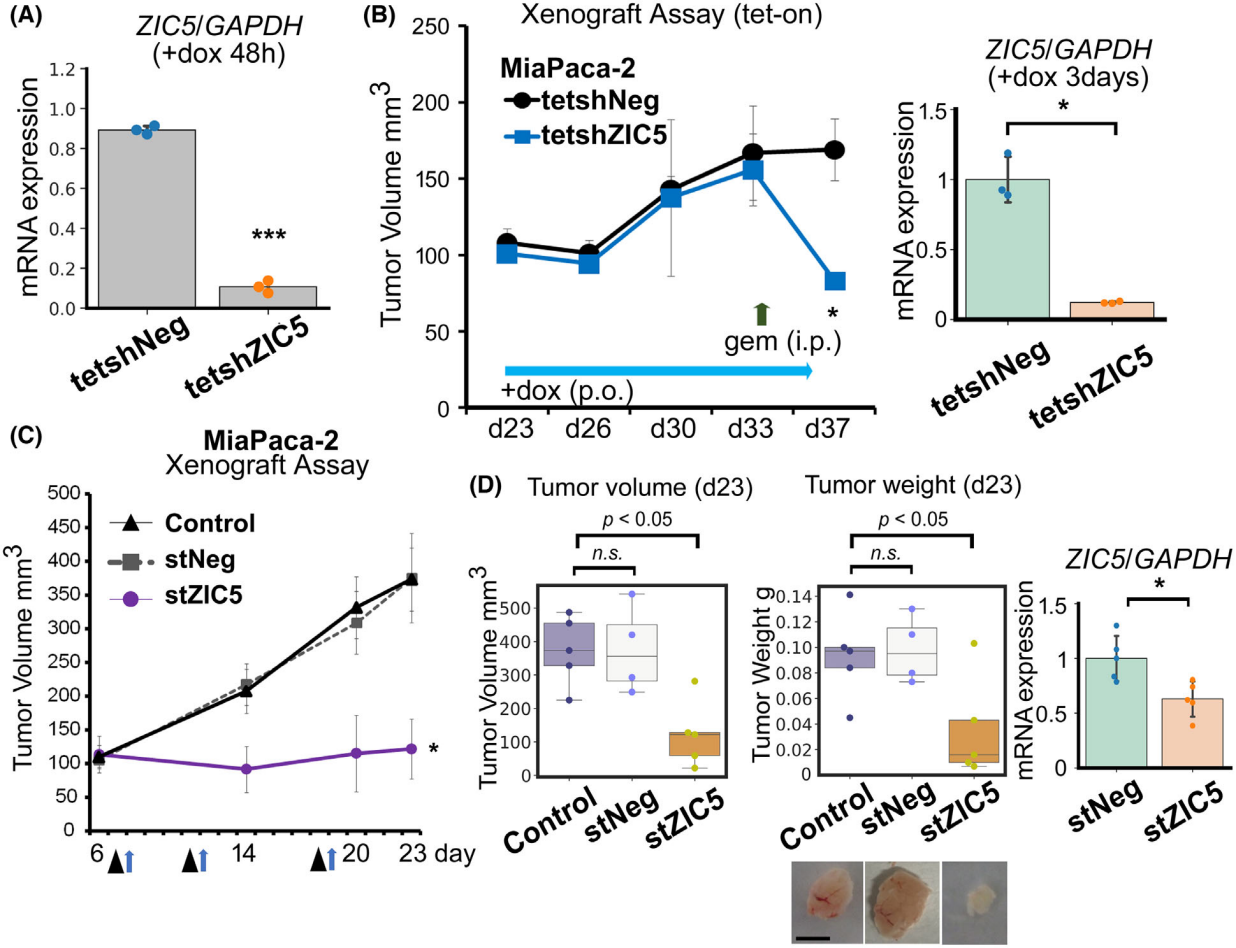
Targeting ZIC5 increases gemcitabine sensitivity and causes PDAC shrinkage *in vivo*. (A) MiaPaca‐2 cells integrated with tetracycline‐inducible (tet‐on) shRNA targeting ZIC5 (tetshZIC5) or nontargeting RNA (tetshNeg) were treated with 0.1 μg·mL^−1^ doxycycline (dox) for 48 h, and the expression of *ZIC5* was quantified using qPCR. Data are presented as mean ± standard deviation (SD). Statistical analysis was performed using the two‐tailed unpaired Student's *t*‐test (biological replicates from three different passages of cells, ***P < 0.001). (B) Tumor volume in nude mice (*n* = 3 for each group) inoculated with MiaPaca‐2 cell lines as indicated and administered with gemcitabine (100 mg·kg^−1^) on day 33 (left). Data are presented as mean ± standard error of the mean (SEM). After 3 days of dox administration, *ZIC5* expression in each xenograft was assessed by qPCR (right). Data are presented as mean ± SD. Statistical analysis was performed using Welch's *t*‐test (*P < 0.05). (C) Tumor volume in nude mice inoculated with MiaPaca‐2 cell lines and treated with atelocollagen complexed with or without stealth siRNA targeting ZIC5 (stZIC5) or negative control (stNeg) on days 6, 11, and 18 (intravenous injections, arrowheads), following gemcitabine treatment (intraperitoneal injections, arrows). Data are presented as mean ± SEM. (D) Tumor volume and tumor weight at day 23 (left). Representative images of xenografts are also shown at the bottom (scale bar = 5 mm). The box plots summarize the medians and values between the 25th and 75th percentiles. Statistical analysis was performed using Dunnett multiple comparison test. *ZIC5* mRNA expression in xenografts was assessed 3 days after stZIC5 injection (right) (*n* = 5 for each group). Data are presented as mean ± SD. Statistical analysis was performed using the two‐tailed unpaired Student's *t*‐test (*P < 0.05). (C, D) Five (Control or stZIC5) or four (stNeg) mice were used for the assessment of tumor volume and weight.

To verify whether *ZIC5* is a suitable therapeutic target for PDAC *in vivo*, we performed xenograft assays in which human PDAC cells (MiaPaca‐2) were transplanted into nude mice. First, we established the cell lines using tetracycline‐inducible (tet‐on) shRNA targeting *ZIC5* (tetshZIC5) or nontargeting RNA (tetshNeg) in MiaPaca‐2 cells (Fig. 1A). These cell lines were subcutaneously transplanted into nude mice and allowed to engraft to a size of approximately 100 mm^3^. Subsequently, shRNA expression was induced by doxycycline administration, and tumor volume was measured. A significant difference between the tumor volumes of mice that received tetshZIC5 cells and those that received tetshNeg cells was not observed until day 33 (Fig. 1B). A significant reduction in *ZIC5* expression in xenografts of tetshZIC5 cells after doxycycline administration was confirmed (Fig. 1B). However, when gemcitabine was administered to all mice from day 33, tumor shrinkage was observed only in the tumors of mice that received tetshZIC5 cells (Fig. 1B). These results demonstrate that targeting *ZIC5* sensitizes PDAC cells to gemcitabine. To further confirm the therapeutic potential of *ZIC5*‐targeting small interfering RNA (siRNA) *in vivo*, MiaPaca‐2 cells were inoculated into nude mice. After the tumor volume was engrafted to approximately 100 mm^3^, atelocollagen [10] with chemically modified siRNA against *ZIC5* (stZIC5), randomized control siRNA (stNeg), or atelocollagen alone (Control) was intravenously injected. Atelocollagen‐mediated systemic delivery is a reliable and safe approach for enhancing siRNA functionality *in vivo* [10]. Subsequently, gemcitabine was injected into all mice. Mice that received stZIC5 exhibited a reduced tumor volume compared to the control or stNeg‐receiving mice (Fig. 1C,D). A significant reduction in *ZIC5* expression in the xenografts of mice treated with stZIC5 was confirmed (Fig. 1D). Notably, two out of five mice in the stZIC5 group showed tumor shrinkage, whereas two showed no growth. These results indicate that *ZIC5* is a suitable therapeutic target for PDAC *in vivo*.

### RNA‐Seq analysis and comprehensive analysis reveal ZIC5‐associated genes

**Fig. 2:**
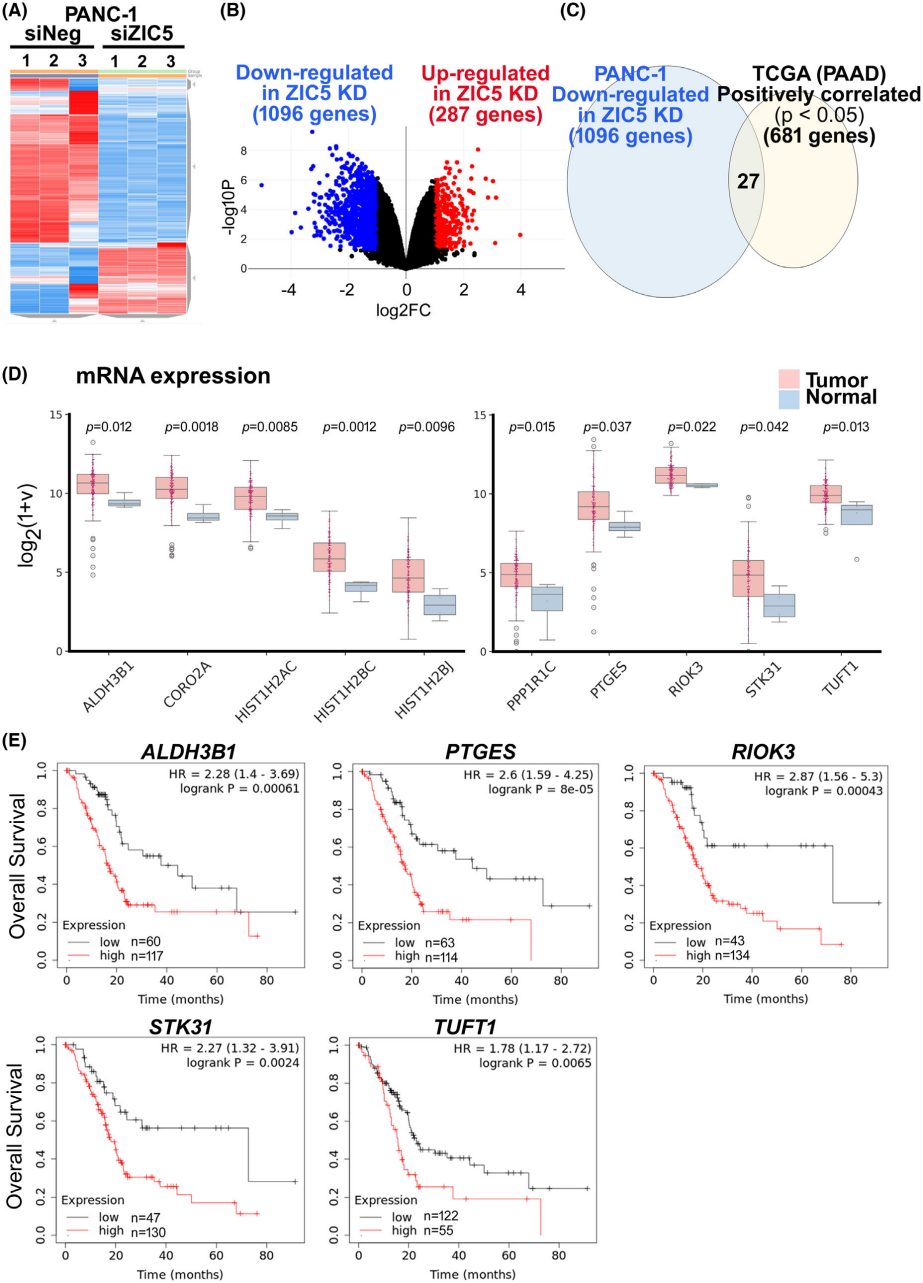
RNA‐Seq analysis and comprehensive analysis reveals ZIC5‐associated genes. (A) The heat map shows the top 2500 genes that had altered expression in response to *ZIC5* knockdown (*n* = 3 for each group). (B) Volcano plot in which fold changes of gene expression were transformed using log_2_ and displayed on the *x*‐axis; *P*‐values were corrected using the Benjamini–Hochberg method, transformed using −log_10_, and displayed on the *y*‐axis. Highlights show 1096 genes downregulated in ZIC5 knockdown (blue) and 287 genes upregulated in ZIC5 knockdown (red). (C) Venn diagram showing the overlap of 27 genes that were both downregulated by *ZIC5* knockdown and positively correlated with *ZIC5* expression in samples from patients with PDAC (cBioPortal analysis with TCGA data (PAAD; *n* = 185)). (D) The expression of 27 genes in primary PDAC samples (*n* = 177) and nontumor samples (*n* = 4) (TCGA). (E) Kaplan–Meier survival curves for patients with pancreatic cancer according to the expression of each gene.

In the present study, we demonstrated that targeting *ZIC5* decreases PDAC resistance to gemcitabine, thereby making treatment more effective. However, the precise molecular mechanisms, including the ZIC5 target genes, are not known. To further investigate the molecular mechanisms underlying ZIC5‐mediated PDAC survival and primary drug resistance, we performed RNA‐Seq analysis (Fig. 2A, Table S1). *ZIC5* knockdown in PANC‐1 cells resulted in greater than twofold changes in the expression of multiple genes, with a significant increase and decrease in the expression of 287 and 1096 genes, respectively (Fig. 2B). Among these, we extracted 27 genes whose expression was positively correlated with that of *ZIC5* in PDAC clinical samples (Fig. 2C, Table S2). Among the 27 genes, 10 were significantly upregulated in PDAC tissues compared to normal tissues (Fig. 2D). Among them, nine genes (*ALDH3B1*, *CORO2A*, *HIST1H2AC*, *HIST1H2BC*, *HIST1H2BJ*, *PTGES*, *RIOK3*, *STK31*, and *TUFT1*) were associated with poor prognosis in patients with PDAC (Fig. 2E, data not shown). The expression of genes thus identified is most likely predominantly regulated by ZIC5 and is related to PDAC progression. Among the nine genes, further investigation focused on the genes encoding proteins with enzymatic activity and proteins secreted outside the cell (*ALDH3B1*, *PTGES*, *RIOK3*, *STK31*, and *TUFT1*).

### ZIC5 positively regulates the expression of *ALDH3B1*, *PTGES*, *RIOK3*, *STK31*, and *TUFT1*

**Fig. 3:**
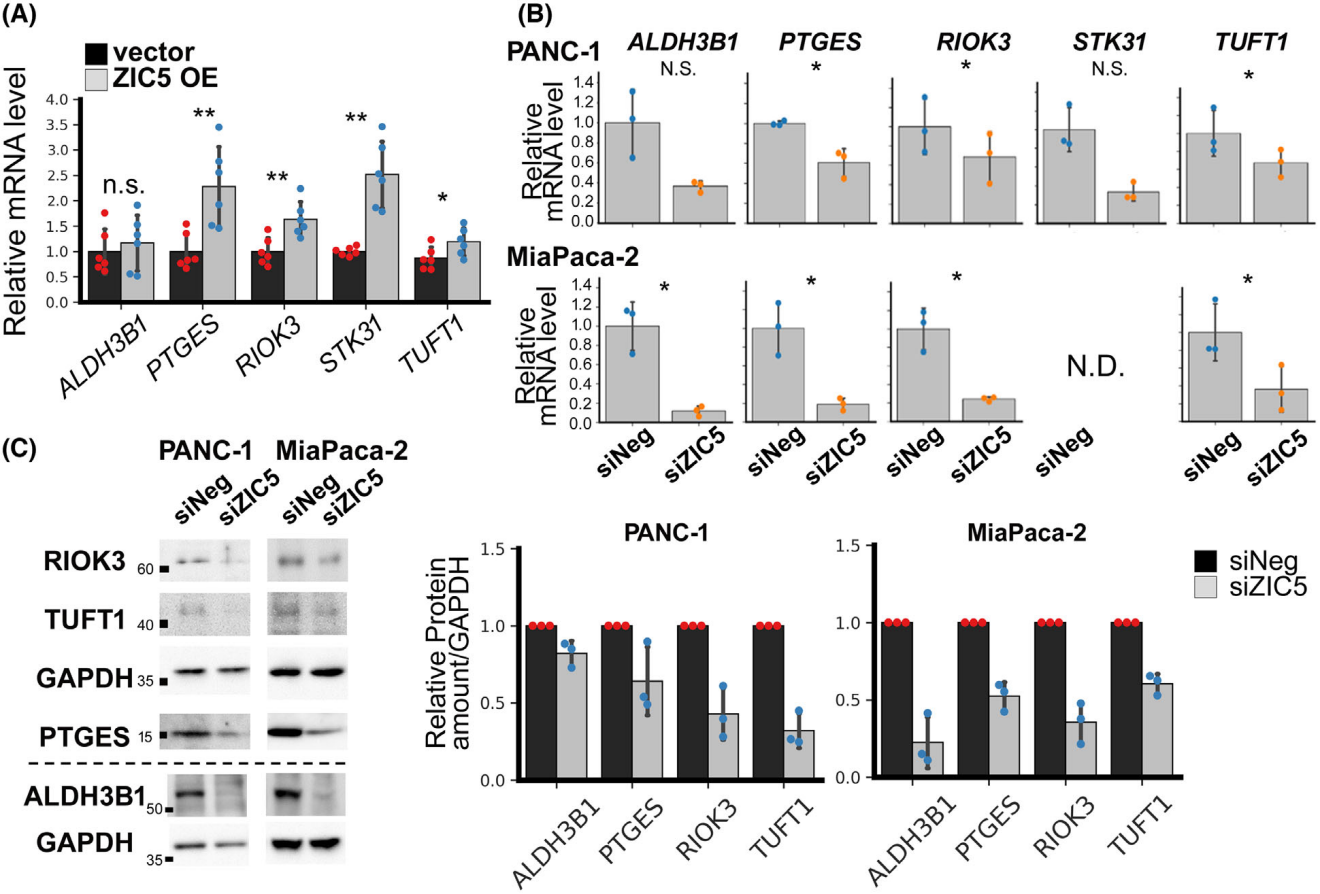
ZIC5 positively regulates the expression of *PTGES*, *RIOK3*, *STK31*, and *TUFT1*. (A) HEK293 cells were transiently transfected with control or *ZIC5* expression vector. After 2 days, cells were harvested, and the expression of each gene was quantified using qPCR (*n* = 6 for each group). The relative expression level was normalized to that of the housekeeping gene *GAPDH*. (B) PANC‐1 and MiaPaca‐2 were transfected with a negative control (siNeg) or *ZIC5* (siZIC5) siRNA. After 2 days, cells were harvested, and the expression of each gene was quantified using qPCR (*n* = 3 for each group). The relative expression level was normalized to that of the housekeeping gene *GAPDH*. (A, B) Statistical analysis was performed using the two‐tailed paired Student's *t*‐test (biological replicates from different passages of cells, *P < 0.05; **P < 0.01). (C) Western blot analysis was performed using the indicated antibodies. Below the dashed line indicates the analysis of a different sample pair. In each blot, the expression levels of each protein level were normalized to that of GAPDH and compared to relative amounts in siNeg‐treated cells (biological replicates from three different passages of cells). (A–C) Data are presented as mean ± SD.

To confirm that the identified genes are regulated by ZIC5, we overexpressed *ZIC5* in HEK293 cells and investigated the expression of these genes. *ZIC5* overexpression resulted in increased expression of *PTGES*, *RIOK3*, *STK31*, and *TUFT1* (Fig. 3A). When *ZIC5* was knocked down, there was a significant decrease in the expression of *ALDH3B1*, *PTGES*, *RIOK3*, and *TUFT1* in both PANC‐1 and MiaPaca‐2 cells. *STK31* expression was decreased by *ZIC5* knockdown in PANC‐1 cells, whereas *STK31* expression was barely detected in MiaPaca‐2 (Fig. 3B). The protein levels of ALDH3B1, PTGES, RIOK3, and TUFT1 were also decreased in both PANC‐1 and MiaPaca‐2 cells following ZIC5 knockdown (Fig. 3C).

### *RIOK3* contributes to the survival of PDAC cells, whereas *ALDH3B1*, *PTGES*, and *TUFT1* contribute to the gemcitabine resistance of MiaPaca‐2

**Fig. 4:**
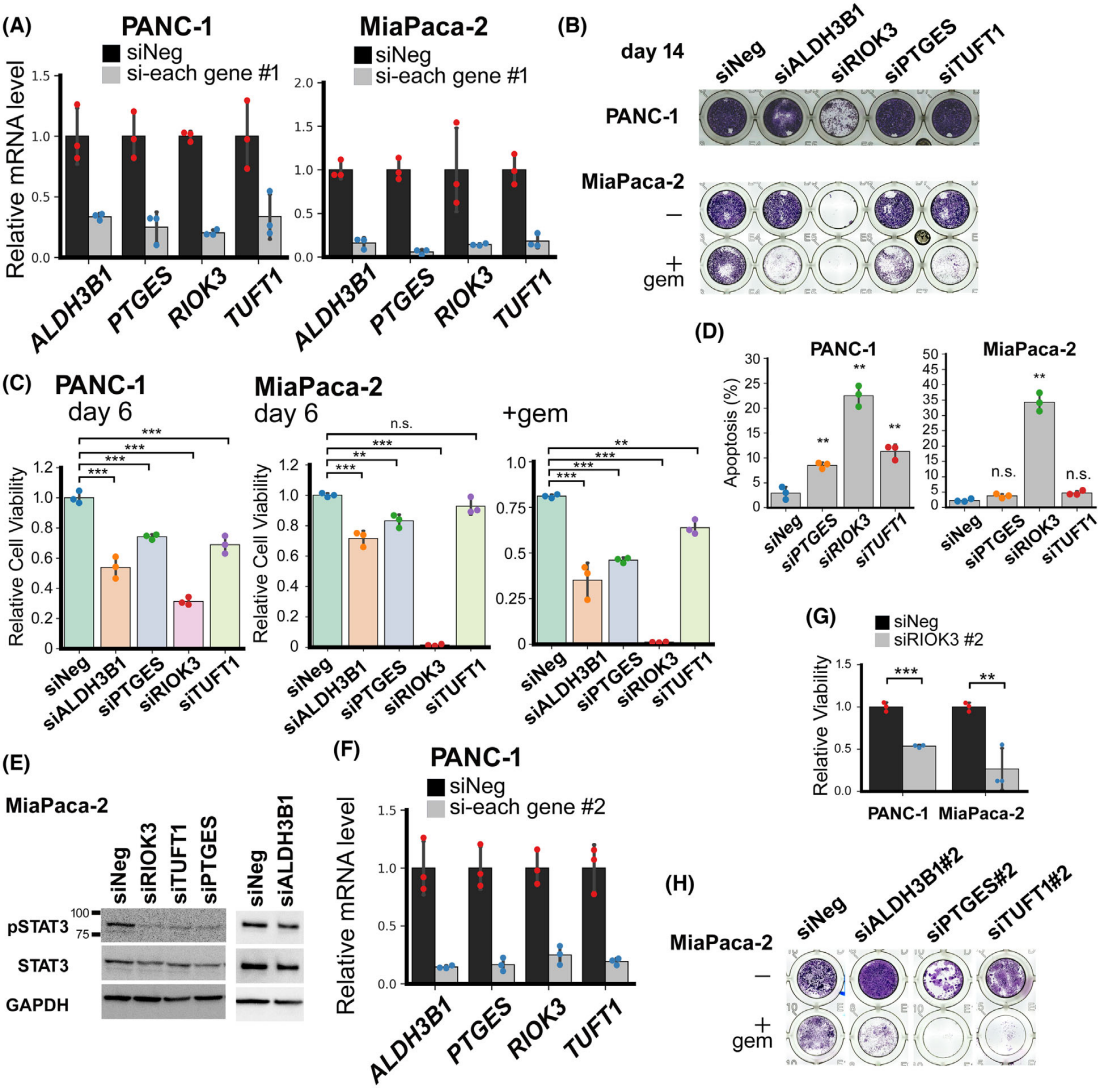
*RIOK3* positively regulates survival of PDAC cells. (A–E) PANC‐1 and MiaPaca‐2 were transfected with a negative control (siNeg) or siRNA for each gene. (A) After 2 days, cells were harvested, and the expression of each gene was quantified using qPCR (biological replicates from three different passages of cells). (B) After 3 days, gemcitabine (0, 50 nm) was added to MiaPaca‐2 cells. After 14 days, the cells were stained with Giemsa solution. Reproducibility was confirmed in three separate independent experiments. (C) Viability of PANC‐1 and gemcitabine‐treated (0, 50 nm) MiaPaca‐2 cells at 6‐day post‐transfection. Statistical analysis was performed using Dunnett multiple comparison test (biological replicates from three different passages of cells, **P < 0.01; ***P < 0.001). (D) After 6 days, the percentage of caspase‐positive cells from three independent experiments was determined. Statistical analysis was performed using Tukey's multiple comparison test (**P < 0.01). (E) After 3 days, cells were assessed using western blotting with indicated antibodies. Reproducibility was confirmed in three separate independent experiments. (F–H) PANC‐1 and MiaPaca‐2 were transfected with a negative control (siNeg) or another siRNA for each gene (#2). (F) The expression of each gene was quantified using qPCR. (G) After 6 days, cell viability was assessed. Statistical analysis was performed using Welch's *t*‐test (biological replicates from three different passages of cells, **P < 0.01; ***P < 0.001). (H) Giemsa staining was performed as in (B). Reproducibility was confirmed in three separate independent experiments. (A, C, D, F, G) Data are presented as mean ± SD.

To elucidate the factors that are important for the survival or drug resistance of PDAC cells, we knocked down *ALDH3B1*, *PTGES*, *RIOK3*, and *TUFT1* using siRNA (Fig. 4A). Because *ZIC5* knockdown induces PANC‐1 eradication by inducing apoptosis and sensitizes MiaPaca‐2 cells to gemcitabine [3], we assessed PANC‐1 viability and MiaPaca‐2 resistance to gemcitabine following knockdown of *ALDH3B*, *PTGES*, *RIOK3*, or *TUFT1*. After 14 days of culture, only *RIOK3* knockdown caused a substantial decrease in cell number of both PANC‐1 and MiaPaca‐2 cells (Fig. 4B), whereas a decrease in viable cells was also observed by knockdown of *ALDH3B*, *PTGES*, or *TUFT1* after 6 days of culture of PANC‐1, MiaPaca‐2, or gemcitabine‐treated MiaPaca‐2 cells (Fig. 4C). After 14 days of culture, knockdown of *ALDH3B*, *PTGES*, or *TUFT1* significantly reduced the growth in low‐dose gemcitabine‐treated MiaPaca‐2 cells (Fig. 4B). Knockdown of *PTGES*, *RIOK3*, or *TUFT1* induced apoptosis in PANC‐1 cells, whereas only *RIOK3* knockdown induced apoptosis in MiaPaca‐2 cells (Fig. 4D). These findings suggest that only RIOK3 knockdown induces a high rate of cell death and thus exhibits antiproliferative effects in long‐term culture (Fig. 4B), whereas knockdown of *ALDH3B*, *PTGES*, or *TUFT1* enhances sensitivity to gemcitabine. Western blotting studies revealed that knockdown of *PTGES*, *RIOK3*, or *TUFT1* reduced STAT3 phosphorylation, which was positively regulated by ZIC5 (Fig. 4E). Testing with other siRNAs for each gene (siRNAs #2) confirmed that *RIOK3* contributes to the survival of PDAC cells (Fig. 4F,G), whereas *ALDH3B1*, *PTGES*, and *TUFT1* contribute to gemcitabine resistance in MiaPaca‐2 (Fig. 4F,H).

### *ZIC5* directly promotes the expression of *RIOK3*

**Fig. 5:**
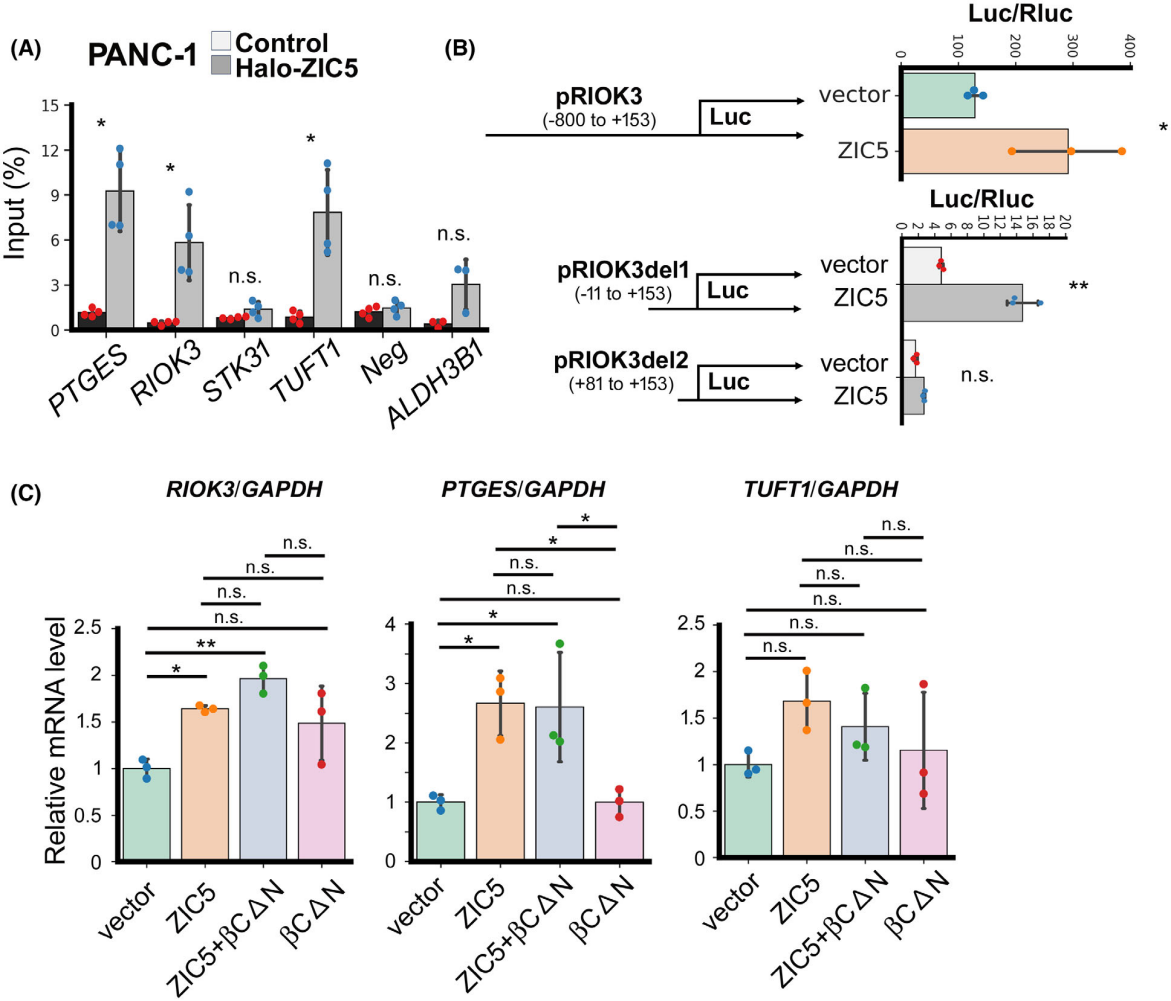
ZIC5 directly promotes the expression of *RIOK3*. (A) Halo‐tagged ZIC5 was overexpressed in PANC‐1 cells, and Halo ChIP assay was performed. The ratio of detected DNA to input is shown. Statistical analysis was performed using Welch's *t*‐test (biological replicates from four different passages of cells, *P < 0.05). (B) The luciferase reporter constructs were transfected with *ZIC5* expression vector or the empty vector. Firefly luciferase activities were normalized to renilla luciferase activities. Statistical analysis was performed using the two‐tailed unpaired Student's *t*‐test (biological replicates from three different passages of cells, *P < 0.05; **P < 0.01). (C) HEK293 cells were transiently transfected with control, *ZIC5*, or β‐cateninΔN expression vector as indicated. After 2 days, cells were harvested, and the expression of each gene was quantified using qPCR. Statistical analysis was performed using Tukey's multiple comparison test (biological replicates from three different passages of cells). (A–C) Data are presented as mean ± SD.

To determine whether ZIC5 directly regulates the expression of downstream genes, a Halo ChIP assay was performed. ZIC5‐pulldown enriched the DNA region near the transcription start sites of *PTGES*, *RIOK3*, and *TUFT1*, whereas the negative control region, *ALDH3B1* and *STK31* region were not enriched (Fig. 5A). Luciferase assay revealed that ZIC5 promoted *RIOK3* promoter activity (Fig. 5B), whereas activation of the *PTGES* and *TUFT1* promoters was not observed (data not shown). Further, promoter assay with the deleted *RIOK3* promoter revealed that ZIC5 regulates *RIOK3* promoter in the −11 to +81 region (Fig. 5B). Next, we assessed whether ZIC5 regulates the expression of *RIOK3*, *PTGES*, and *TUFT1* in association with β‐catenin [6, 9]. The expression of *RIOK3*, *PTGES*, and *TUFT1* was not affected by overexpression of β‐cateninΔN, which is a stable construct of β‐catenin [11] (Fig. 5C).

### *RIOK3* is one of the important survival factors, which is directly regulated by ZIC5 in PDAC

**Fig. 6:**
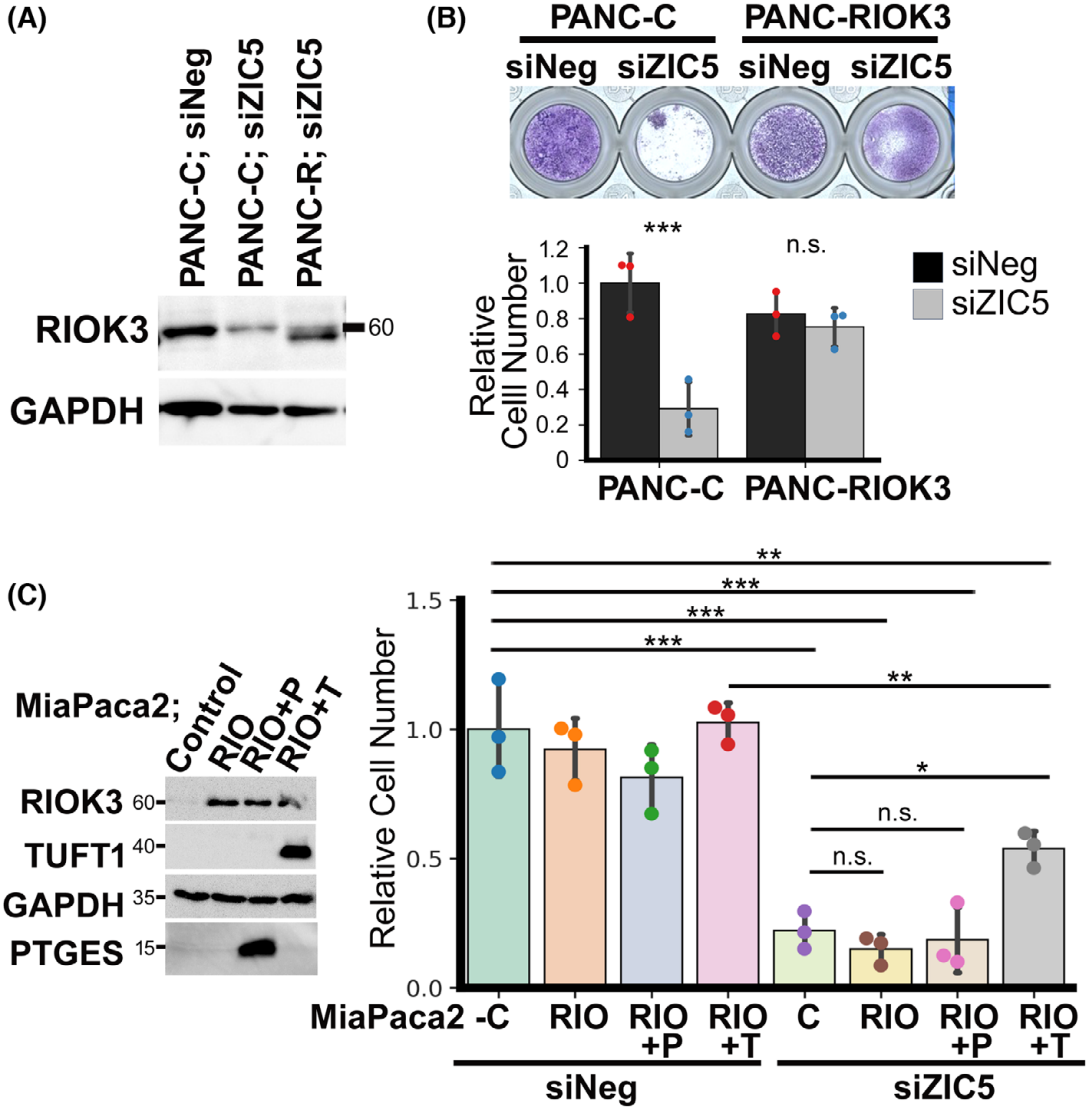
Stable overexpression of RIOK3 rescued the cell number reduction induced by *ZIC5* knockdown in PANC‐1 cells. (A) PANC‐1 stably overexpressing RIOK3 (PANC‐R) and the control cell (PANC‐C) were assessed using western blotting with the indicated antibodies. Reproducibility was confirmed in three separate independent experiments. (B) PANC‐1 cell lines were transfected with a negative control (siNeg) or siRNA for *ZIC5* (siZIC5). After 14 days, the cells were stained with Giemsa solution. Relative cell number was quantified. Statistical analysis was performed using the two‐tailed unpaired Student's *t*‐test (biological replicates from three different passages of cells, ***P < 0.001). (C) MiaPaca‐2 stably overexpressing RIOK3 (RIO), PTGES (P) and/or TUFT1 (T) were assessed using western blotting with the indicated antibodies. These cell lines were transfected with siNeg or siZIC5. After 6 days, cell number was determined. Statistical analysis was performed using Tukey's multiple comparison test (biological replicates from three different passages of cells, *P < 0.05, **P < 0.01, ***P < 0.001). (B, C) Data are presented as mean ± SD.

To elucidate whether *RIOK3* is an essential factor directly regulated by ZIC5 for PDAC cell survival, PANC‐1 and MiaPaca‐2 cell lines stably overexpressing RIOK3 were established (Fig. 6A,C). Stable overexpression of RIOK3 rescued the decrease in cell number induced by *ZIC5* knockdown in PANC‐1 cells but not in MiaPaca‐2 cells (Fig. 6A–C). A stable cell line in which PTGES or TUFT1 was co‐overexpressed with RIOK3 was established in MiaPaca‐2 cells (Fig. 6C). When TUFT1 was co‐overexpressed with RIOK3, the decrease in cell number induced by *ZIC5* knockdown was partially rescued (Fig. 6C). These results demonstrate that *RIOK3* is an important survival factor for PDAC downstream of ZIC5; however, other *ZIC5*‐downstream factors are also important in different contexts.

## Discussion

In this study, we demonstrated through *in vivo* experiments that ZIC5 is a promising therapeutic target for PDAC treatment. However, to develop drugs targeting ZIC5, it is important to elucidate the molecular mechanisms by which ZIC5 promotes PDAC cell survival.

To date, the molecular mechanisms and direct targets of ZIC5 in PDAC remain unclear. This study revealed that *RIOK3* is a ZIC5 downstream gene that is important for survival signaling in PDAC cells. RIOK3 is a member of the right open reading frame (RIO) family, which is characterized by the presence of the RIO kinase domain and is critical for the maturation of the 40S small ribosomal subunit [12, 13]. RIOK3 is highly expressed in PDAC and is associated with poor prognosis. RIOK3 is amplified and promotes invasive activity through activation of the small G protein Rac [14]. Furthermore, RIOK3 binds to and stabilizes FAK to promote the invasion of PDAC cells [15]. In our experiments, stable overexpression of RIOK3 (even in combination with TUFT1) did not restore the effect of *ZIC5* suppression in MiaPaca‐2 cells, suggesting that other ZIC5 downstream genes also contribute to pancreatic cancer survival. It is highly possible that ZIC5 is involved in the expression of histones (Fig. 2), which were not analyzed in this study, and is involved in cell proliferation.

Recently, the efficacy of cancer immunotherapy has been reported in various cancers. However, the efficacy of the immune checkpoint inhibitors currently in clinical use is limited for PDAC. One possible reason for this is that the immunosuppressive microenvironment is composed of a variety of immunosuppressive cells such as regulatory T cells (Tregs), tumor‐associated macrophages (TAMs), and myeloid‐derived suppressor cells (MDSCs) [16]. In the present study, we found that ZIC5 also regulates PTGES expression, although its direct regulation could not be confirmed. PTGES is a critical enzyme that catalyzes the conversion of prostaglandin H2 into a prostaglandin E2 (PGE2), which suppresses antitumor immunity and fuels tumor‐promoting inflammation [16]. Database analysis has reported higher expression of PTGES in pancreatic tumors than in normal pancreatic tissues. Moreover, PTGES expression was associated with the downregulation of the major histocompatibility complex pathway and negatively correlated with CD8^+^ T‐cell activation markers [17]. These results suggest that PTGES regulates the immune microenvironment of PDAC. Therefore, targeting ZIC5 may more effectively shrink PDAC tumors in mice with a wild‐type immune system.

Zhao *et al*. [9] showed that ZIC5 regulates glucose metabolism in colorectal cancer cell lines by regulating *SLC2A1* expression. In our study, several members of the solute carrier family were upregulated or downregulated by *ZIC5* knockdown in PANC‐1 cells; however, *SLC2A1* expression was not significantly altered (Table S1). Recently, ZIC was shown to be SUMOylated in response to WNT signaling which increases ZIC5 transcriptional activation while reducing ZIC5/TCF co‐repression during neural crest development [18]. Thus, the cellular context (e.g., post‐transcriptional regulation and co‐factors) may render a variety of transcriptional regulatory targets of ZIC5. In this study, we investigated the ZIC5 transcriptional activity with or without Wnt/β‐catenin signaling by co‐overexpressing β‐cateninΔN with ZIC5. However, no significant relationship was observed (Fig. 5C). The identification of factors that cooperate with ZIC5 to regulate transcription remains challenging.

In this study, we demonstrate that targeting *ZIC5* increases gemcitabine sensitivity and causes PDAC shrinkage *in vivo*. Additionally, we identified that *ALDH3B1*, *PTGES*, and *TUFT1*, the downstream genes of *ZIC5*, contribute to the gemcitabine resistance of MiaPaca‐2 (Fig. 4). In the development of drugs that target ZIC5, it is expected that the expression levels of these downstream genes will serve as indicators of effective inhibition of ZIC5.

## Conclusions

In conclusion, our results indicate that ZIC5 is an attractive therapeutic target for the treatment of PDAC and that some of the target genes identified in this study can be used as markers of ZIC5 activation or suppression.

## Materials and methods

### Cell culture

The PANC‐1 (RRID: CVCL_0480) and MiaPaca 2 (RRID: CVCL_0428) cell lines were obtained from the American Type Culture Collection (Manassas, VA, USA). All cell lines were authenticated using short tandem repeat (STR) profiling within the last 3 years. All experiments were performed using mycoplasma‐free cells. Cells were maintained at 37 °C in a 5% CO_2_ humidified atmosphere in RPMI 1640 medium (Invitrogen, Carlsbad, CA, USA) supplemented with 10% fetal bovine serum.

### Establishment of stable cell lines

To obtain stable cell lines, we introduced the tetracycline‐inducible (tet‐on) shRNA targeting ZIC5 (tetshZIC5). The target sequence, 5′‐gattcgaggctgtgacaaa‐3′, was integrated into the EcoRI/AgeI site of tet‐pLKO‐puro plasmid (#21915; Addgene, Watertown, MA, USA). HEK293 cells were then transfected with tet‐pLKO‐puro containing both pCMV‐VSV‐G (RIKEN RDB04392) and psPAX2 (#12260; Addgene), and the culture medium was incubated with MiaPaca‐2 cells with polybrene (5 μg·mL^−1^) for 24 h. Stable cell lines were obtained using selection with puromycin (2 μg·mL^−1^).

To obtain stably overexpressing cell lines, the ORFs of *RIOK3*, *PTGES*, and *TUFT1* were amplified and cloned into the pMX‐IP or pMX‐IN vector. PLAT‐A cells were transfected with the vector, the culture medium was incubated with PANC‐1 or MiaPaca‐2 cells with polybrene, and the cells were selected using puromycin (2 or 4 μg·mL^−1^) or G418 (1000 μg·mL^−1^).

### RNA isolation, cDNA synthesis, and quantitative real‐time PCR

These assays were performed as described previously [11, 19]. Quantitative real‐time PCR (qPCR) was performed using the primers listed in Table S3. *GAPDH*, which encodes the housekeeping protein glyceraldehyde phosphate dehydrogenase, was used as an internal control for the normalization of transcript levels.

### Animal experiments

For xenograft assays, 2.5 × 10^6^ cells resuspended in PBS (0.05 mL) and Matrigel (0.05 mL) (Corning, Corning, NY, USA) were subcutaneously inoculated into the flanks of 5‐week‐old female BALB/c *nu/nu* mice (CLEA, Tokyo, Japan). Mice were maintained in a temperature‐ and humidity‐controlled environment (23 ± 1 °C, 55 ± 5% humidity) with a 12/12 h light/dark cycle. One week after tumor inoculation, individual mice were intravenously injected with 200 μL of atelocollagen (Koken, Tokyo, Japan) complexed with 4 nmol of Stealth RNAi™ (siRNA) targeting *ZIC5* (*n* = 5), randomized negative control stealth RNA (stNeg) (*n* = 4) (Thermo Fisher Scientific, Carlsbad, CA, USA), or PBS as the control (*n* = 5). Gemcitabine (100 mg·kg^−1^) was intraperitoneally administered. To induce tet‐on shRNA expression, the mice (*n* = 3, each group) were provided with water containing doxycycline (1 mg·mL^−1^; TaKaRa, Shiga, Japan). The tumor volume was calculated according to the formula *V* = 1/2 (*A* × *B* ^2^), where *A* and *B* represent the largest and smallest tumor dimensions, respectively. All animal experiments were approved by the Institutional Ethics Committee and performed in compliance with the Guidelines for Laboratory Animal Research of the Tokyo University of Pharmacy and Life Sciences (Tokyo, Japan) (approved number; L24‐02). Ethical endpoints were defined by the ethical endpoint policy in accordance with the Guidelines for Proper Conduct of Animal Experiments (Science Council of Japan).

### Small interfering RNA and plasmid transfection

Negative control siRNA and siRNA targeting *ZIC5* used in this study have been previously described [4]. siRNA targeting *ALDH3B1* (#1; SI00294217, #2; SI03104983) *PTGES* (#1; SI03035816, #2; SI00069594), *RIOK3* (#1; SI02223403, #2; SI02223396), and *TUFT1* (#1; SI04247271, #2; s227658) were purchased from Qiagen (Hilden, Germany) or Ambion (Austin, TX, USA). Transient transfections with siRNA were performed using Lipofectamine RNAiMAX (Invitrogen) according to the manufacturer's protocol. In each experiment, the total amount of transfected siRNA was adjusted using the relevant negative control siRNA. Plasmid transfection was performed as described previously [4, 19].

### RNA‐sequencing analysis

Total RNA was purified and *ZIC5* knockdown (FC < 0.1) was confirmed using qPCR. Library preparation, sequencing, and subsequent data processing were performed by GENEWIZ (Tokyo, Japan). Total RNA was quantified using the Qubit RNA Assay and TapeStation RNA ScreenTape. cDNA libraries for RNA sequencing were established from 500 ng of total RNA using the NEBNext Poly(A) mRNA Magnetic Isolation Module (New England Biolabs, Ipswich, MA, USA) for selecting poly‐A mRNA, followed by strand‐specific library preparation using MGIEasy RNA Directional Library Prep Set V2.0 (MGI Tech, Shenzhen, China). The adapter sequences used for library preparation were as follows:

Forward: AAGTCGGAGGCCAAGCGGTCTTAGGAAGACAA;
Reverse: AAGTCGGATCGTAGCCATGTCGTTCTGTGAGCCAAGGAGTTG.

The resulting double‐stranded library fragments were further processed into single‐stranded circular DNA (sscDNA). The sscDNA libraries were quantified and used to generate DNA nanoballs (DNBs). The DNBs were then sequenced using the DNBSEQ‐G400 platform (MGI Tech) according to the manufacturer's instructions. To remove technical sequences (including adapters, polymerase chain reaction (PCR) primers, or fragments thereof) and quality of bases lower than 20, pass filter data in fastq format were processed using cutadapt (V1.9.1). RNA‐Seq reads were aligned to a reference human genome (hg38/GRCh38) using the hisat2 software (v2.0.1). Raw data files were deposited in the GEO database (https://www.ncbi.nlm.nih.gov/geo/query/acc.cgi?acc=GSE183860). Transcripts per kilobase million (TPM) normalization was used to perform differential expression analyses.

### Cell viability and apoptosis assays

Cell viability was assessed using Cell Counting Kit‐8 (Dojindo, Kumamoto, Japan), and apoptosis was assessed using CellEvent Caspase‐3/7 Green Detection Reagent (Invitrogen) and Hoechst33342 (Dojindo) as previously described [3].

### Western blot analysis

Western blotting was performed as previously described [11, 20]. Primary antibodies are listed in Table S3. GAPDH was used as a loading control.

### Halo ChIP assay

Cells were transfected with or without the Halo‐tagged ZIC5 expression vector, fixed with 1% paraformaldehyde for 10 min at 25 °C, and the reactions were stopped by the addition of glycine to a final concentration of 125 mm for 10 min. Cells were processed according to the manufacturers' protocol using HaloLink™ Resin (Promega, Tokyo, Japan). The protein–DNA complexes were then reverse‐crosslinked by heating for 6 h at 65 °C, followed by proteinase K treatment. The resulting DNA was purified using a PCR Purification Kit (Qiagen) and analyzed by qPCR using specific primers (Table S3).

### Luciferase reporter assay

The luciferase reporter construct pRIOK3‐Luc and deletion mutants were constructed by amplifying the *RIOK3* promoter region with primers (Table S3) and subcloned into the XhoI–NcoI region of the pGL4.10‐luc2 vector (Promega). Cells were cotransfected with the luciferase reporter plasmid and a pGL4.70 internal control plasmid (Promega) using polyethylenimine. Luciferase activity was measured using a Dual‐luciferase Reporter Assay System (Promega).

### Statistical analysis

The expression values of PDAC genes were obtained from RNA sequence data in The Cancer Genome Atlas (TCGA) (http://cancergenome.nih.gov/), and statistical analysis was performed using a two‐tailed Mann–Whitney *U* test. Survival analysis was performed using a Kaplan–Meier plotter [21]. Other statistical analyses were performed using the r statistical software package (v. 4.0.3). Data are presented as mean ± standard deviation (SD) in bar graphs, unless otherwise indicated. The box plots summarize the medians and values between the 25th and 75th percentiles. Significant differences were determined using the statistical tests indicated in the individual figure legends. *P* < 0.05 was considered statistically significant.

## Conflict of interest

The authors declare no conflict of interest.

## Author contributions

RS contributed to conception, design, and development of methodology. RS, YK, MO, ST, and YA contributed to data acquisition, data analysis, and interpretation. All authors contributed to writing, review, and/or revision of the manuscript. AY, KH, and KF contributed to administrative, technical, and material support. RS contributed to study supervision. The work reported in this article was performed by the authors unless otherwise specified.

## Supporting information


**Table S1.** Differential gene expression analysis of RNA sequence data of PANC‐1 cells transfected with siNeg or siZIC5.

**Table S2.** Positively correlated genes to ZIC5 in PAAD samples.

**Table S3.** Resource table.

## Data Availability

RNA‐seq data that support the findings of this study are openly available in the NCBI Gene Expression Omnibus (https://www.ncbi.nlm.nih.gov/geo/query/acc.cgi?acc=GSE183860). Other data supporting the findings of this study are available upon request from the corresponding author.
